# Flood impacts on urban road connectivity in southern China

**DOI:** 10.1038/s41598-022-20882-5

**Published:** 2022-10-07

**Authors:** Ruitao Zhou, Hang Zheng, Yueyi Liu, Guanti Xie, Wenhua Wan

**Affiliations:** 1 School of Environment and Civil Engineering, Dongguan University of Technology, Dongguan, 523808 Guangdong China; 2 Dongguan Shigu Sewage Treatment Co., Ltd, Dongguan, 523808 China

**Keywords:** Environmental sciences, Hydrology, Natural hazards

## Abstract

Effective measures to improve road accessibility during storms are required as traffic congestion caused by storm floods increasingly constrains the efficiency of urban commuting. However, flood impacts on urban road connectivity are not yet well assessed due to inaccurate simulation of flood processes in urban areas where high-resolution data for drainage networks and gauged hydrological data are insufficient. Thus, this study assesses flood impacts on road network connectivity in an urban area of southern China through joint modeling of 1-D hydrodynamic processes in drainage networks and 2-D flood inundation processes on roads using MIKE Urban and MIKE 21. High-resolution DEM images of 5 m and a drainage network of 5635 pipelines were used for urban hydrological simulation. Flood depths were gauged for model calibration and validation by recruited volunteers in the context of citizen science. The results show that road network connectivity decreases as rainfall increases. More than 40% of road connectivity is lost in the study area when a 1-in-100-year return period rainfall occurs. The study results can help to inform more adaptive strategies for local flood control. The study methods are also applicable to improving urban hydrological modeling in broader regions.

## Introduction

Floods are causing increasing damage and losses to human society in this era of global climate change^1–3^. The negative effects of floods are more intense in urban areas because of the intensive land use changes^4,5^. Over the past several decades, urban floods have considerably increased in frequency and severity due to changes in urban hydrological processes caused by climate and land use changes^6,7^. Flood control plays a critical role in urban security management^8^, especially in developing countries experiencing rapid urbanization, as in China^9–11^.

Flooding has significant impacts on various aspects of human society^12,13^. Understanding the complex nature of flood losses provides a foundation for effective flood management^14,15^. Generally, there are two types of losses from floods: direct and indirect^16,17^. Direct damages are those that occur due to physical contact of flood water with humans, property, or other objects^16^. Indirect damages are wide-ranging, including interruption of traffic that may lead to disruptions in enterprise production and financial losses^18^. In urban areas, indirect losses from floods may be more widespread, because high-rise buildings and other places of refuge could reduce people’s direct contact with floodwaters, while infrastructure in low-lying areas such as roads, water and gas pipelines, underground subway stations as well as parking lots are flooded, also causing substantial indirect losses^19–21^.

Storm floods that occur due to heavy rainfall are the major type of urban flooding^22^. Extreme rainfall combined with acceleration of runoff yield caused by increased impervious surfaces in urban areas has caused urban floods to occur increasingly quickly^23,24^. Urban infrastructure may be flooded in a very short time. As an example, a storm-induced flood devastated Zhengzhou, a city in the central region of China, in July 2021, with maximum 24-h rainfall reaching 663.9 mm^25^. Water depth on major flooded roads surpassed 1 m after just several hours, causing flood damage to 400,000 cars, about 40.9 billion Yuan in losses. Moreover, the water depth in some subway tunnels exceeded a typical person's chest height in just dozens of minutes, directly claiming the lives of those with insufficient time to escape. Low-lying traffic infrastructure has become a hotspot of inundation losses due to insufficient time for people or cars to evacuate during rapid flooding^25^.

Models have been developed to improve urban flood management through simulation of urban hydrological processes^26,27^. For example, Bhattacharjee, et al.^28^ established a storm water management model to analyze flood peak flow and the flooding extent of Bhubaneswar City, India, using elevation, slope, land use/land cover and storm water drain infrastructure data. Jamali, et al.^29^ developed RUFIDAM to rapidly estimate flood extent, depth, and associated damage. RUFIDAM was tested in Melbourne, and the resulting prediction of flood extent and accumulated damage cost showed acceptable accuracy. In addition, simulation time was reduced compared to MIKE FLOOD. Quan, et al.^30^ analyzed the impact of land use/cover change on surface runoff and evaluated flood risk in Shanghai through a simplified urban waterlogging model. These authors found that surface runoff depth increased by 13.19 mm from 1994 to 2006 due to urbanization.

These studies contribute to improved flood management in urban areas, especially for urban road networks. Transportation infrastructure is a key component of the economic growth and development of urban areas^31–34^. However, such infrastructure may comprise the major assets affected by inundation, causing not only infrastructure damage but also transportation disruption^35^. Existing studies assess the vulnerability and accessibility of roads during floods by jointly applying the rainfall–runoff model, the 2-D hydrodynamic model, and road network analysis^36,37^. For example, Klipper, et al.^38^ evaluated the accessibility of road networks to hospitals during floods in Jakarta using urban hydraulic models. Katya, et al.^18^ analyzed the impacts of flooding on traffic in St Maarten, the Netherlands, by integrating a flood model (MIKE Flood) and a traffic model (SUMO). Singh, et al.^39^ found that more than 40% of road length across the Indian road network becomes impassable when a 1-in-100-year rainfall event occurs, via simulation of road flooding processes.

Road inundation maps in the context of various rainfall events can be generated using methods from the above studies to identify roads at flood risk. However, knowledge gaps remain in that an overall or holistic assessment method for evaluating the performance of inundation maps is lacking. This causes difficulty in comparing different road inundation maps under various flood scenarios. An integrated index, such as one representing changes in road network connectivity across different flood scenarios, is then required to further inform urban flood management by comparing the overall effects of floods.

Accordingly, a comprehensive method for road flood analysis is proposed in this study by jointly applying 2-D flood modeling and road inundation and road network connectivity assessment. Dongguan City, Guangdong Province, China, is adopted as the case study area. The main goals include: (a) assessing flood impacts on urban road connectivity in the case study area; and (b) identifying the relationship between the return periods of rainfall and road network connectivity. This paper proceeds as follows. “Methods” section introduces our method, and “Study area and data” section introduces the data and the data processing. “Results” and “Discussion” sections present the results and discussion. Finally, “Conclusion” section sets forth our conclusions.

## Methods

### Methodological framework

**Figure 1:**
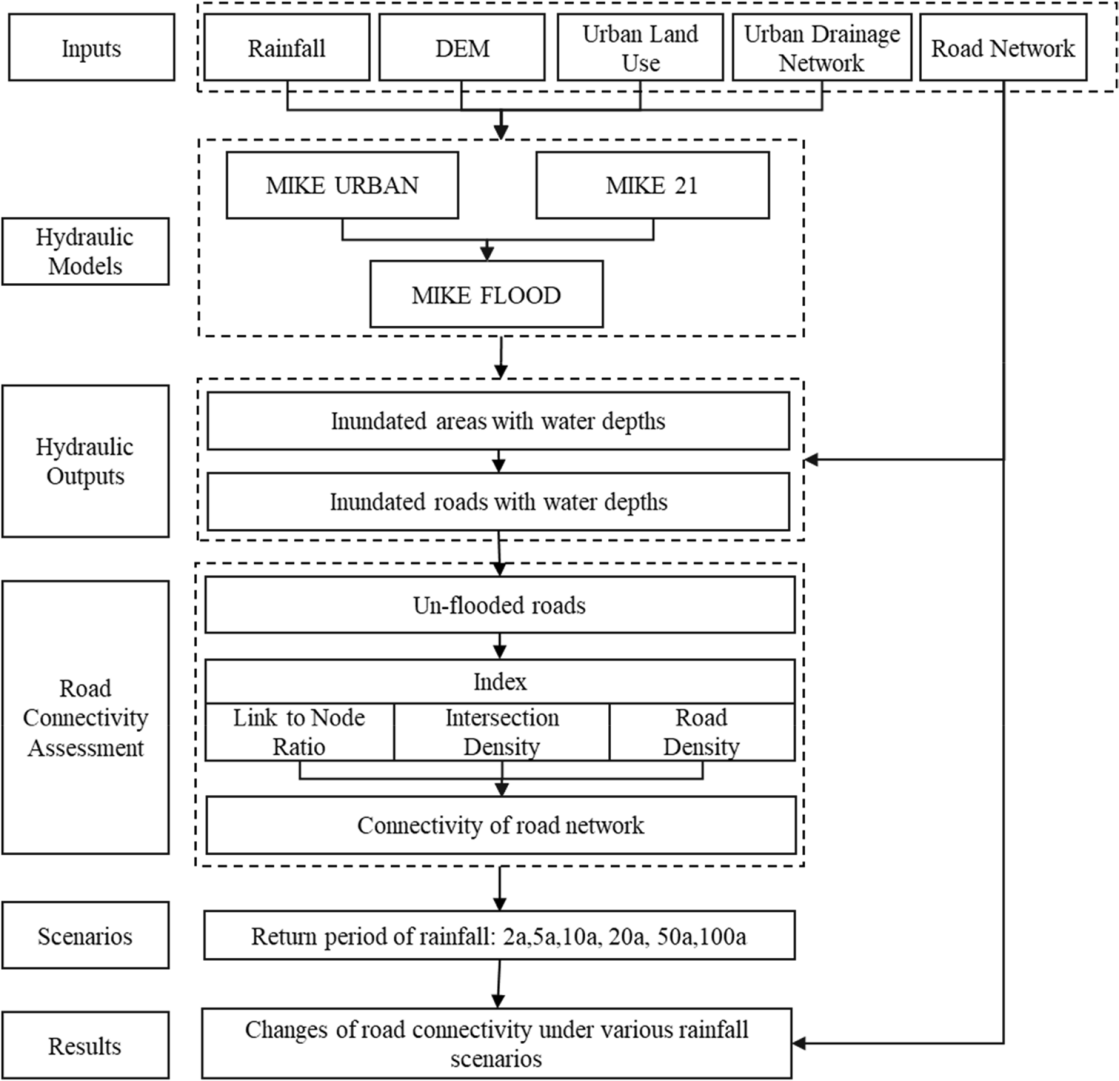
Methodological framework. This graph was generated with Microsoft PowerPoint version 2206(https://www.office.com/).

The methodological framework is shown in Fig. 1, in which three calculation steps are represented to assess connectivity changes in the flooded road network under various rainfall scenarios. In the first step, the inundated areas and their water depths in the case area are calculated by simulating urban rainfall–runoff processes using hydrologic and hydrodynamic models. Then, inundated road segments are extracted from inundated urban areas based on the road network map in the study area. It is assumed that inundated segments with water depths over 0.15 m are unavailable for vehicles and pedestrians to pass through. The connectivity of the road network during floods is calculated from the un-flooded roads, which are defined as road segments for which the inundated water depth is less than 0.15 m. The un-flooded road network is obtained by removing road segments with water depth over 0.15 m from the original road network. Finally, in the third step, the connectivity of the road network is assessed using three indicators: Link-Node Ratio (*LNR*), Intersection Density (*ID*), and Road Network Density (*RND*), based on previous studies on road network connectivity^40–42^.

### Hydraulic models

The software packages of MIKE Urban, MIKE 21, and MIKE Flood, developed by the Danish Institute of Hydraulics, are used to model the rainfall–runoff and flood processes in the case area.

#### MIKE urban

MIKE Urban is applied to calculate the volume of surface runoff by deducting the infiltrated and drained water underground from the rainfall amount. Data on urban land use and drainage networks are used in MIKE Urban. The hydraulic processes in the drainage pipelines are simulated according to the 1-D Saint–Venant equations^43^, which are as follows:

1 $$\frac{\partial Q}{{\partial x}} + \frac{\partial A}{{\partial t}} = 0,$$

2 $$\frac{\partial Q}{{\partial t}} + \frac{{\partial \left( {\alpha \frac{{Q^{2} }}{A}} \right)}}{\partial x} + gA\frac{\partial h}{{\partial x}} + \frac{gQ\left| Q \right|}{{C^{2} AR}} = 0,$$

where Eqs. (1) and (2) represent mass conservation and momentum conservation, respectively. *Q*, *x*, *A*, and *t* represent the flow (m^3^/s), distance (m), area of cross-section (m^2^), and calculation time (s), respectively. *g*, *C*, and* R* represent the acceleration of gravity (m/s^2^), Chézy coefficient (m^1/2^·s^-1^), and hydraulic radius (m), respectively.

#### MIKE 21

MIKE 21 is used to calculate inundated areas and water depths of surface runoff through hydraulic simulation based on 2-D continuity and momentum equations^44^, as follows:

3 $$\frac{\partial h}{{\partial t}} + \frac{{\partial h\overline{u}}}{\partial x} + \frac{{\partial h\overline{v}}}{\partial y} = hS,$$

4 $$\begin{aligned} \frac{{\partial h\overline{u}}}{\partial t} + \frac{{\partial h\overline{u}^{2} }}{\partial x} + \frac{{\partial h\overline{uv} }}{\partial y} & = f\overline{v}h - gh\frac{\partial \eta }{{\partial x}} - \frac{h}{{\rho_{0} }}\frac{{\partial p_{a} }}{\partial x} - \frac{{gh^{2} }}{{2\rho_{0} }}\frac{\partial p}{{\partial x}} + \frac{{\tau_{sx} }}{{\rho_{0} }} - \frac{{\tau_{bx} }}{{\rho_{0} }} \\ & \quad - \frac{1}{{\rho_{0} }}\left( {\frac{{\partial S_{xx} }}{\partial x} + \frac{{\partial S_{xy} }}{\partial y}} \right) + \frac{\partial }{\partial x}\left( {hT_{xx} } \right) + \frac{\partial }{\partial y}\left( {hT_{xy} } \right) + hu_{s} S, \\ \end{aligned}$$

5 $$\begin{aligned} \frac{{\partial h\overline{v}}}{\partial t} + \frac{{\partial h\overline{uv} }}{\partial x} + \frac{{\partial h\overline{v}^{2} }}{\partial y} & = - f\overline{u}h - gh\frac{\partial \eta }{{\partial y}} - \frac{h}{{\rho_{0} }}\frac{{\partial p_{a} }}{\partial y} - \frac{{gh^{2} }}{{2\rho_{0} }}\frac{\partial p}{{\partial y}} + \frac{{\tau_{sy} }}{{\rho_{0} }} - \frac{{\tau_{by} }}{{\rho_{0} }} \\ & \quad - \frac{1}{{\rho_{0} }}\left( {\frac{{\partial S_{yx} }}{\partial x} + \frac{{\partial S_{yy} }}{\partial y}} \right) + \frac{\partial }{\partial x}\left( {hT_{xy} } \right) + \frac{\partial }{\partial y}\left( {hT_{yy} } \right) + hv_{s} S, \\ \end{aligned}$$

where Eq. (3) represents the continuity equation in which *h* represents the water depth of the wave (m). *h* = *d* + *η*, where *d* is the steady state water depth (m), and *η* is the height between the wave surface and still water level (m). *t* represents the calculation time step (s), and $\overline{u}$ and $\overline{v}$ are the flow velocities in the *x* and *y* directions (m/s), respectively. *S* is the water flow from source points. Equations (4) and (5) are momentum equations in the *x* and *y* directions, in which *f* is the Coriolis force coefficient. *g*, $\rho , \rho_{0}$, and $p_{a}$ are the acceleration of gravity (m/s^2^), water density (kg/m^3^), reference water density (kg/m^3^), and atmospheric pressure (Pa), respectively. $s_{xx} , s_{xy}$, $s_{yx} , s_{yy}$ are stresses from wave radiation (N). $T_{xx} , T_{xy}$, $T_{yx} , T_{yy}$ are shear stresses (N). $u_{s} , v_{s}$ are flow velocities at the source point (m/s).

#### MIKE flood

MIKE Flood is applied as a module that couples the 1-D hydrodynamic model (MIKE Urban) and the 2-D model (MIKE21) for joint simulations of rainfall–runoff and flood processes^45^.

The flow rate and water level in the drainage pipe were calculated by 1-D hydrodynamic model (MIKE Urban) using the land surface runoff as the inflow to the pipe. The surface runoff was obtained by hydrological simulations in the sub- catchments which were divided according to the spatial distribution and the ground elevations of the pipes. Based on these, the overflow from the pipe to the ground was calculated using the model of orifice outflow in MIKE Flood. Finally, the inundated water depth of an area near the overflow point was calculated through 2-D hydrodynamic model (MIKE21) using the terrain data of the area.

### Road connectivity assessment

Connectivity refers to the ability to complete links between nodes in transport networks^46,47^. A higher level of connectivity plays a critical role in improving the accessibility of a transport system, which could provide more effective routes to destinations^48^. Connectivity is measured by evaluating the intensity of connections between road segments using the number of links, intersections, and dead ends of a road network^46^. An integrated indicator for assessing road connectivity in the case area is established through normalizing and averaging three sub-indexes: the *LNR*, *ID*, and *RND*, which are widely used for road connectivity assessment in previous studies^49–51^. The *LNR* is defined as an index equal to the number of links divided by the number of nodes within a road network, as shown in Eq. (6). Links are defined as roadway or pathway segments between two nodes. Nodes are intersections or the end of a road^49,52^. *ID* is defined as the number of intersections per unit area^53^, as shown in Eq. (7). *RND* is defined as the linear road length per area of the network^41^, as shown in Eq. (8). A more comprehensive indicator, the Road Network Connectivity Index (*RNCI*), is established through integrating these three indexes, as shown in Eq. (9). According to previous studies, a larger *RNCI* represents stronger connectivity of the road network.

6 $$LNR_{i} = \frac{{NL_{i} }}{{NP_{i} }},$$

7 $$ID_{i} = \frac{{NP_{i} }}{S},$$

8 $$RND_{i} = \frac{{LR_{i} }}{S},$$

9 $$RNCI_{i} = \left[ {\frac{{LNR_{i} }}{{LNR_{max} }} + \frac{{ID_{i} }}{{ID_{max} }} + \frac{{RND_{i} }}{{RND_{max} }}} \right]/3,$$

where *LNR*_*i*_, *ID*_*i*_, and *RND*_*i*_ represent the Link-Node Ratio, Intersection Density, and Road Network Density, respectively, of un-inundated road segments under rainfall event *i*. *NL*_*i*_, *NP*_*i*_, and *LR*_*i*_ are the number of roadway segments, the number of intersections, and the total length of roadway segments, respectively. *S* represents the total area of the road network. *RNCI*_*i*_ is the connectivity of the road network during rainfall event *i*. *LNR*_*max*_, *ID*_*max*_, and *RND*_*max*_ represent the maximum value of *LNR*_*i*_, *ID*_*i*_, and *RND*_*i*,_ respectively.

### Consent to participate

The authors declare that all the listed authors participate in this article.

## Study area and data

### Study area

Shatian Town, with an area of 111.5 km^2^, in Guangdong Province, China, was selected as the case study area. The mean annual rainfall of the area is 1861.6 mm/a. Shatian Town is a highly urbanized area located between Guangzhou and Shenzhen, which are the two biggest metropolises in southern China. As a transportation junction connecting the two metropolises, Shatian Town plays an important role in the commuting of millions of people every year. However, in recent years, it has been facing traffic congestion caused by frequent storm floods. Assessing road connectivity changes during storms is required to implement more efficient and adaptive traffic management in the area.

### Data and processing

#### Rainfall

Rainfall data were obtained from a meteorological station (latitude 22.91, longitude 113.87) in the case area. The meteorological station was established by Dongguan University of Technology; it has measured rainfall for the case area at 30-min intervals since 2018. The gauged rainfall from 17:00 to 22:00 on May 31, 2021 was used for model calibration and validation.

#### Urban drainage network

The urban drainage network map was obtained from the relevant municipal department of the case area. After data processing and cleaning using CAD and ArcGIS software, a drainage network consisting of 5635 pipes with a total length of 198 km, 5487 nodes, and 269 drainage outlets was selected.

#### DEM

DEM data with a spatial resolution of 5 m were applied. This high-resolution DEM image was generated from 147,640 elevation points measured on-site by the local municipal department in 2019. Instead of using satellite DEM data, high-resolution ground observation data were used; this is helpful in increasing the accuracy of the hydraulic simulation.

#### Urban land use

The land use data for the case area at a spatial resolution of 1 m were obtained by reading the boundary data of the object from remote sensing images of Google Earth.

The types of objects including buildings, roads, grasslands, and water bodies were then recognized by manually visual identification.

#### Road network

**Figure 2:**
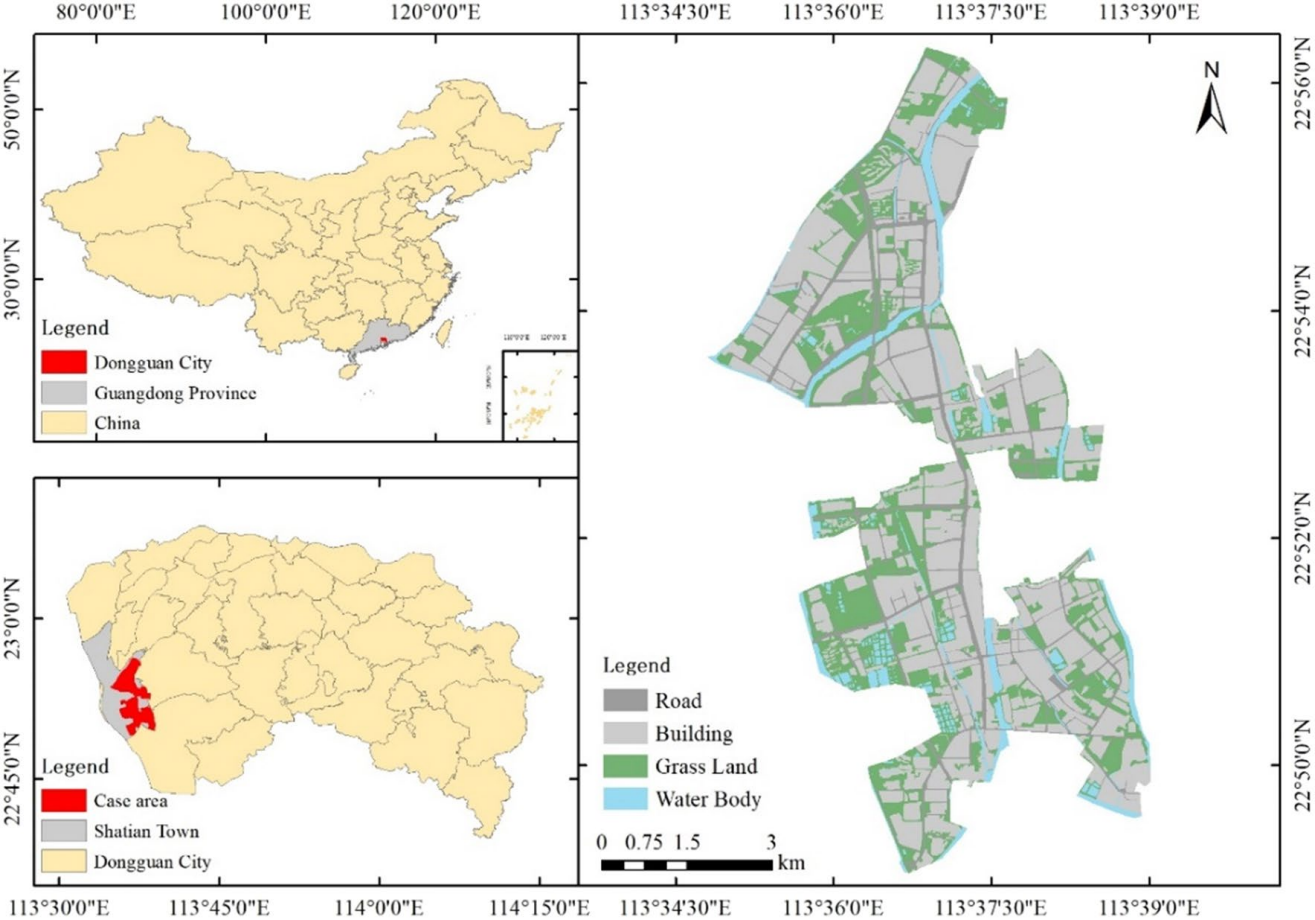
Location of and land uses in the case area. This map was generated with ArcMap Version 10.0. (https://www.esri.com/en-us/arcgis/products/arcgis-desktop/overview).

**Figure 3:**
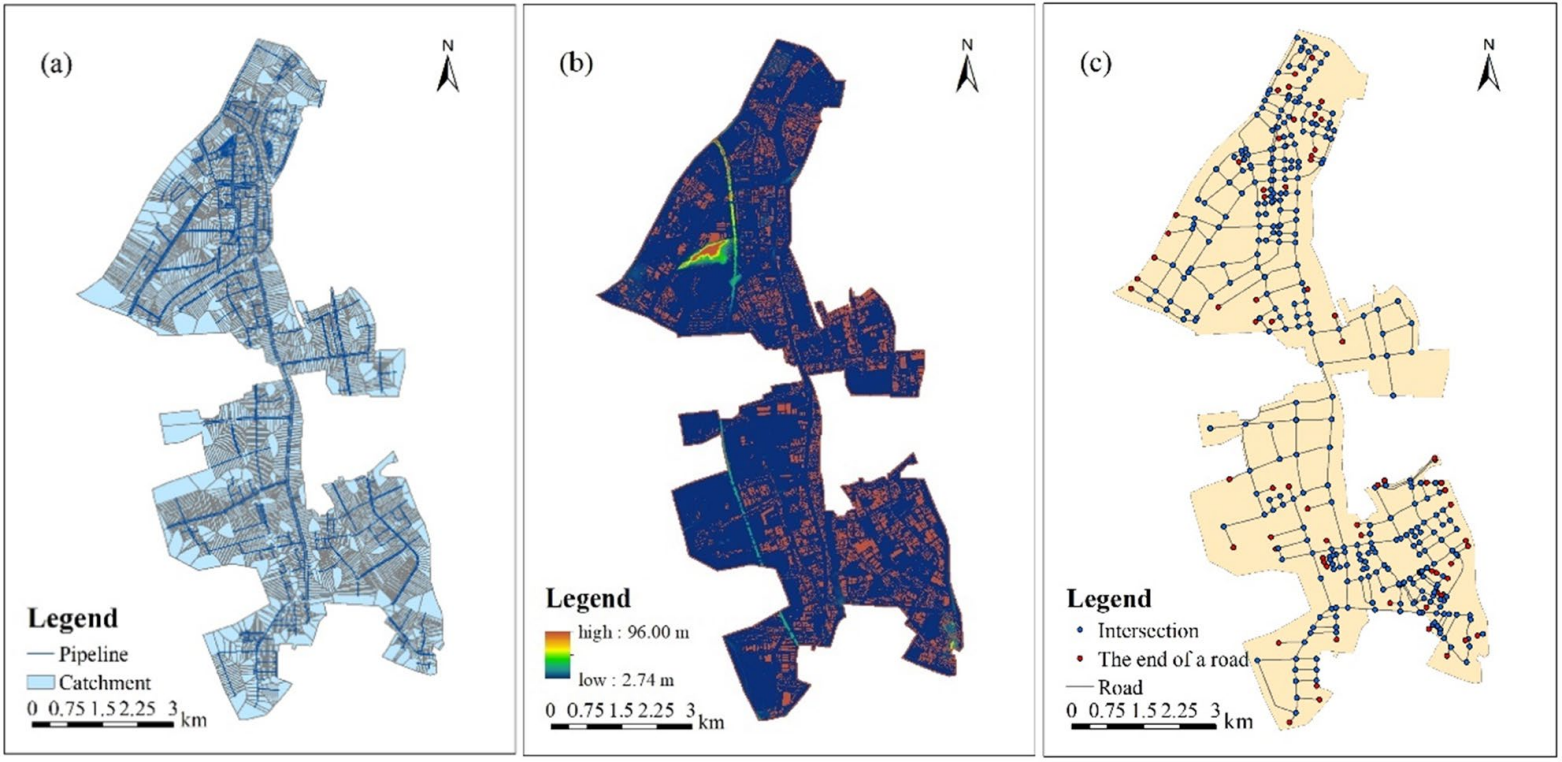
Data for modeling simulation: (**a**) urban drainage network; (**b**) DEM data of case area; (**c**) road network. This map was generated with ArcMap Version 10.0. (https://www.esri.com/en-us/arcgis/products/arcgis-desktop/overview).

Road data was obtained from the OpenStreetMap (https://www.openstreetmap.org/) rather than Google Earth because the geographic information of the roads can be extracted directly from the OpenStreetMap without efforts of manually visual identification. The road data was compared and calibrated with the Google Earth’s map to ensure its spatial consistency with the land use data. The length of roads in the study area is 153.47 km, or 3.57 km per square kilometer. The data on land use, drainage network, DEM, and area road network are shown in Figs. 2 and 3.

### Climate scenarios

Six scenarios of rainfall processes consisting of rainfall return periods of 2a, 5a, 10a, 20a, 50a, and 100a were adopted. The return period of a rainfall event refers to the average time interval between the occurrence of a rainstorm with intensity greater than or equal to a particular value^54^. A longer return period means a larger amount of precipitation.

**Figure 4:**
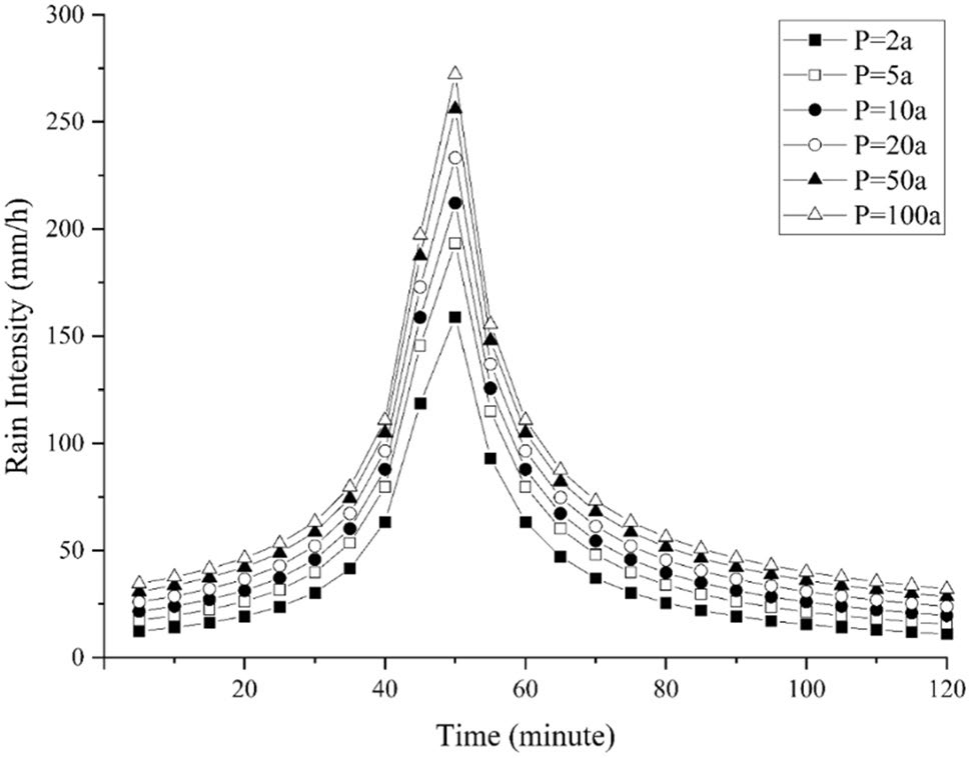
Two-hour rainfall process of adopted return periods. This graph was generated with OriginPro 2021 (Learning Edition)(https://www.originlab.com/).

Rainfall duration of 2 h and a rain peak coefficient of 0.367 were used to graph rainfall events through a Chicago rain type generator^55^ based on the "Rainstorm Intensity Formula and Calculation Chart (2016)" of the local area according to previous studies^56^. The rainfall scenarios are shown in Table 1 and Fig. 4.

**Table 1:** Return period and corresponding total rainfall over 2 h.

Return period (in years)	Total rainfall (2 h) in mm
2a	77.87
5a	99.57
10a	112.92
20a	127.19
50a	142.13
100a	153.09

## Results

### Results of model calibration and validation

**Figure 5:**
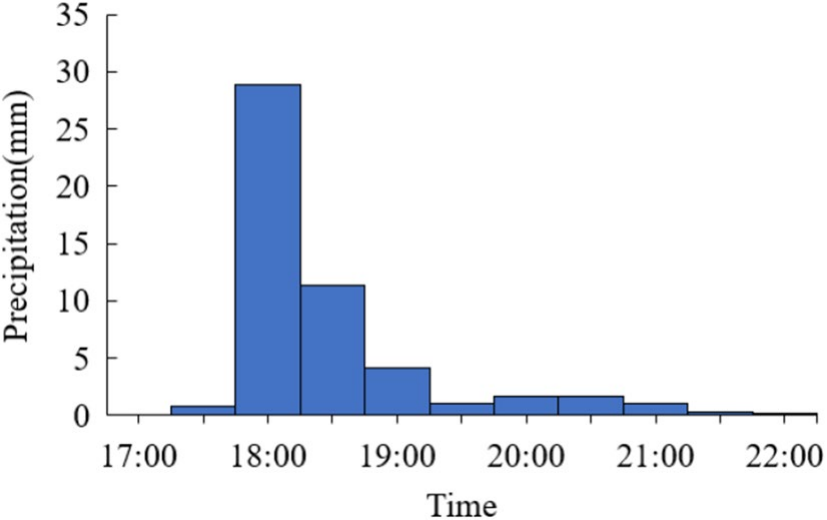
Rainfall event in the case area on May 31, 2021. This graph was generated with OriginPro 2021 (Learning Edition)(https://www.originlab.com/).

**Figure 6:**
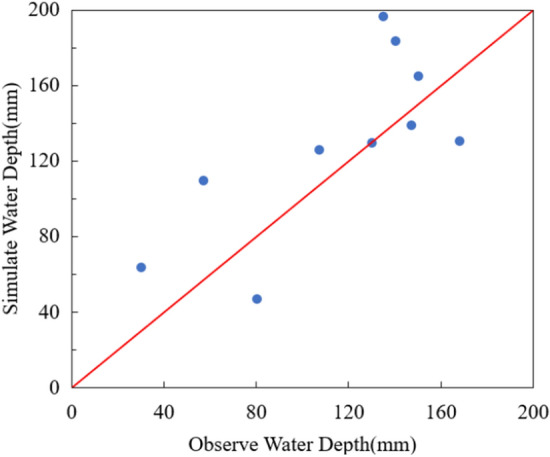
Comparison of observed and simulated water depth. This graph was generated with OriginPro 2021 (Learning Edition)(https://www.originlab.com/).

The models were calibrated by comparing the calculated and observed water depths during a rainfall event in the case area. Water depths at 10 key locations were measured manually from 17:00 to 22:00 on May 31, 2021, during which the total rainfall over 5 h reached 51.44 mm. The rainfall process is shown in Fig. 5. As there are no hydrographic monitoring stations in the case area, flood depths were gauged manually using rulers and on-site photos. Residents were recruited to provide photos or videos of flooding near them, from which the changes in water depths at residents’ locations during a rainfall event were estimated. Based on this, the maximum water depths during rainfall were adopted for model calibration and validation. The observed and simulated maximum water depths at the 10 locations in the case area are shown in Fig. 6.

### Changes in flooded areas under various rainfall scenarios

Changes in flooded areas in the case region across the above rainfall scenarios are shown in Table 2. Note that the flooded area increased as rainfall increased from the return period of 2a to 100a. During the 100a rainfall, the flooded area reached 20.42 km^2^, accounting for 47.51% of the total area of the case region. Moreover, data on flooded areas with various water depths are provided in Table 2, also showing significant positive relationships with rainfall.

**Table 2:** Changes in flooded area with various water depths.

Return periods of rainfall	2a	5a	10a	20a	50a	100a
Total flooded area (km^2^)	17.20	18.57	19.20	19.69	20.13	20.42
Flooded area with water depth beyond 0.15 m (km^2^)	1.06	2.06	2.73	3.49	4.60	5.83
Flooded area with water depth beyond 0.30 m (km^2^)	0.06	0.12	0.20	0.27	0.40	0.58
Maximum depth of the whole flooded area (m)	1.110	1.136	1.145	1.190	1.236	1.268

The flooded areas are divided into two parts according to water depth, including areas with an average water depth above 0.15 m and 0.30 m. During the 2a rainfall, the area with a water depth over 0.15 m accounted for 6.16% of the total flooded area, and the area with a water depth over 0.30 m accounted for 0.34% of the total flooded area. During the 100a rainfall, the area with water depths beyond 0.15 m and 0.30 m reached 28.55% and 2.84% of the total flooded area, respectively. Water depth thresholds of 0.15 m and 0.30 m were adopted in this study to represent details of the flood changes, as the passage of pedestrians and vehicles may be significantly affected in areas with water depths beyond these values according to previous studies^57^. However, the results show that the area with water depth beyond 0.30 m occupied a small part of the region.

Further, maximum flood depths in the study area varied from 1.110 to 1.268 m as rainfall increased. This insignificant change in maximum flood depth implied that areas with higher water depths were concentrated in a small region consisting of hotspots significantly affected by urban flooding.

**Figure 7:**
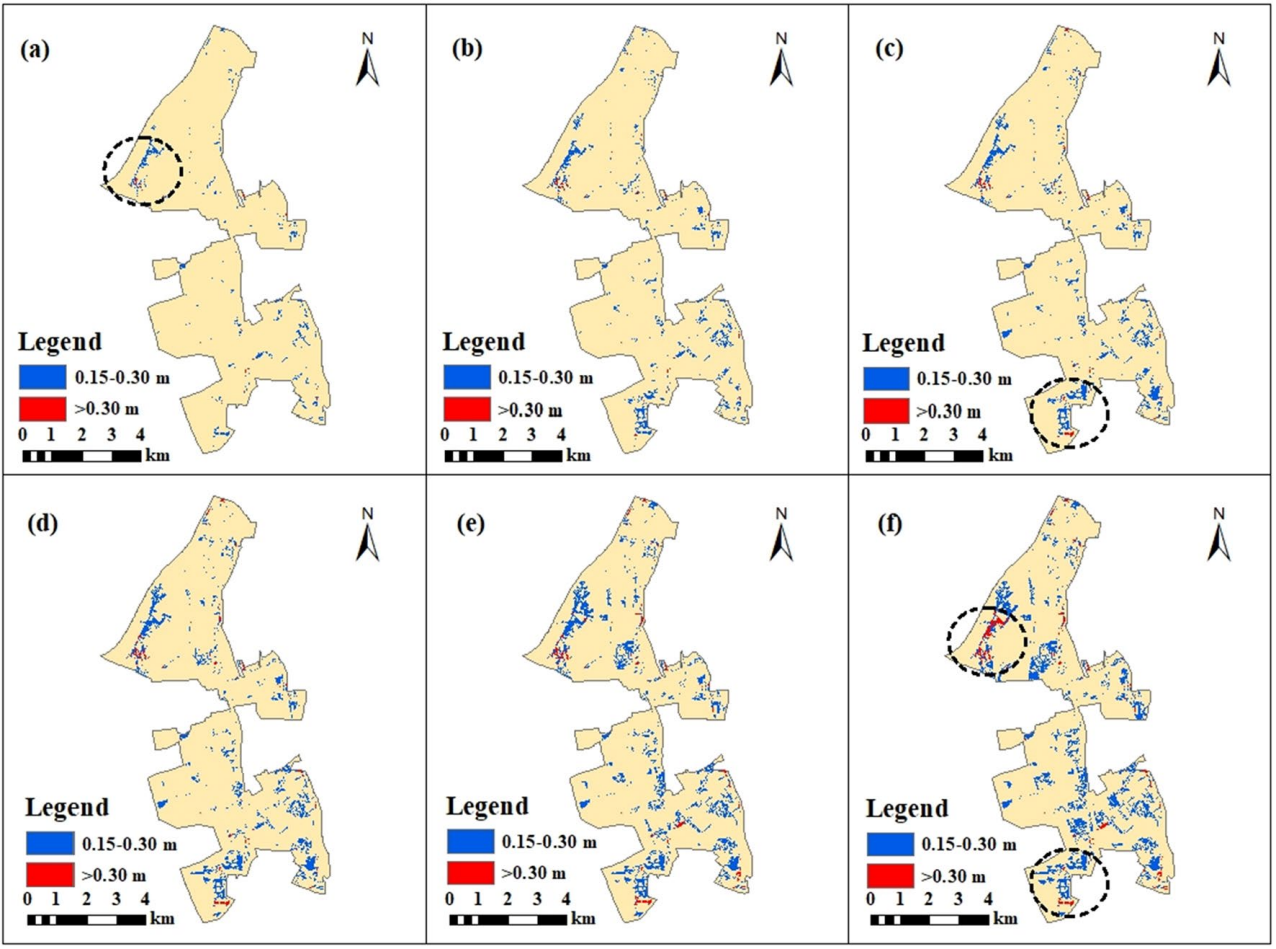
Flooded area under different rainfall return periods including (**a**) Return Period = 2a; (**b**) Return Period = 5a; (**c**) Return Period = 10a; (**d**) Return Period = 20a; (**e**) Return Period = 50a; (**f**) Return Period = 100a. This map was generated with ArcMap Version 10.0. (https://www.esri.com/en-us/arcgis/products/arcgis-desktop/overview).

The spatial distribution of maximum water depths is depicted in Fig. 7. Two hotspots of flooding can be recognized in the northwest and the southern part of the region, indicated by the dotted line. At the first hotspot in the northwest, the area with over 0.30 m flood depth emerged during the 2a rainfall return period and increased as rainfall increased. The maximum water depth of this area ranges from 0.527 to 0.605 m across the rainfall scenarios. For the second hotspot in the south, water depths began to exceed 0.30 m from the 10a rainfall period. The maximum water depth increases to 0.841 m under the 100a rainfall.

**Figure 8:**
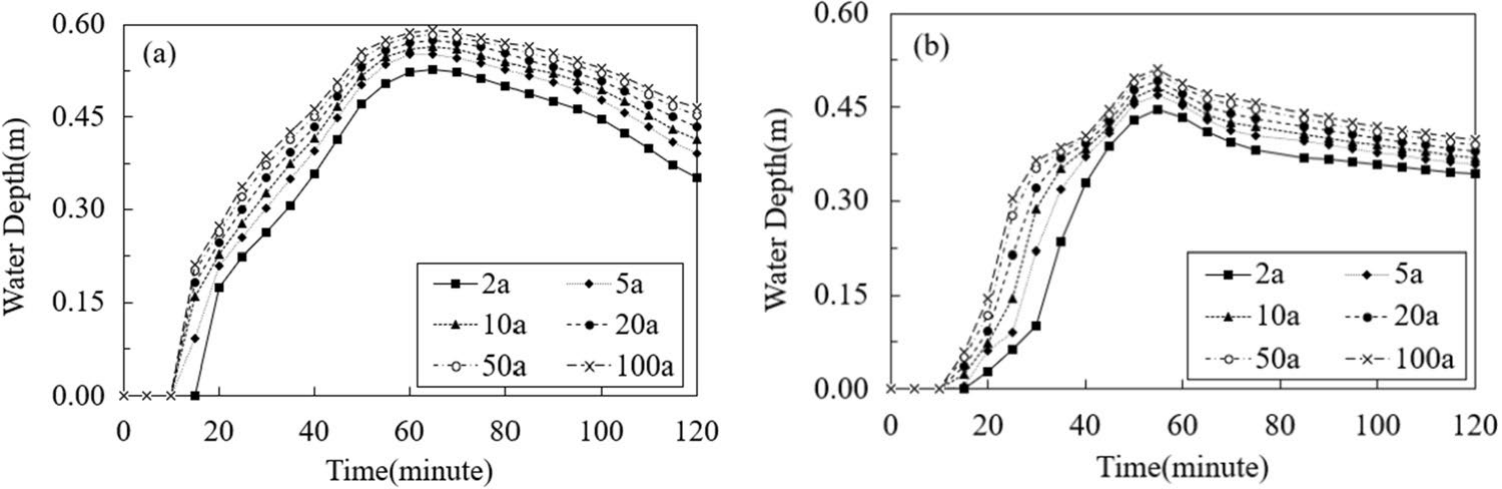
Changes of water depths at (**a**) the northwest spot and (**b**) the southern spot. This graph was generated with OriginPro 2021 (Learning Edition)(https://www.originlab.com/).

Figure 8 shows the changes of water depths at the northwest and southern hotspots across various return periods of the rainfall. As shown in the figure, the water depths of both the spots increased and reached the peaks exceeding 40 cm in the first hour of the modeling. After that, the water depths decreased. In addition, there were higher levels of inundated water at those spots when the return periods of rainfall increased.

### Impact of urban flooding on the road network

**Figure 9:**
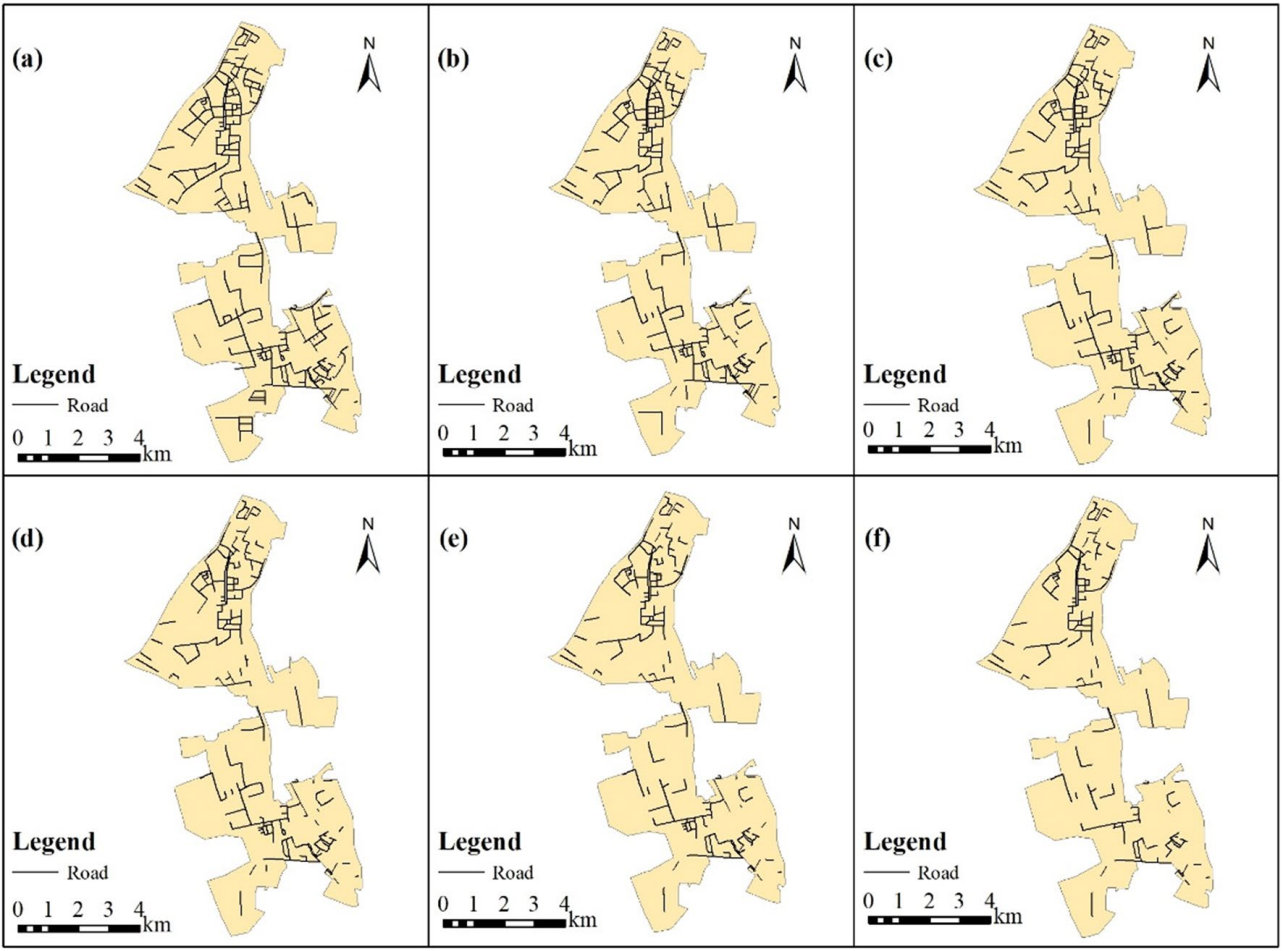
Un-flooded road network under different rainfall scenarios: (**a**) Rainfall Return Period = 2a; (**b**) Rainfall Return Period = 5a; (**c**) Rainfall Return Period = 10a; (**d**) Rainfall Return Period = 20a; (**e**) Rainfall Return Period = 50a; (**f**) Rainfall Return Period = 100a. This map was generated with ArcMap Version 10.0. (https://www.esri.com/en-us/arcgis/products/arcgis-desktop/overview).

The characteristics of the un-flooded road network under various rainfall scenarios are shown in Table 5. The un-flooded road network was shown in Fig. 9. The characteristics of the un-flooded road network under various rainfall scenarios are shown in Table 3. Un-flooded roads are defined as road segments for which inundated water depth is less than 0.15 m. The un-flooded road network, shown in Fig. 9, was then obtained by removing road segments with water depth over 0.15 m from the original road network.

**Table 3:** Changes in road network characteristics across various rainfall scenarios.

Characteristics of the road network	Original road network	Un-flooded road segments under different return periods of rainfall					
		2a	5a	10a	20a	50a	100a
Number of road segments	511	390	343	317	285	259	218
Length of roads (km)	155.6	105.9	91.1	81.5	72.9	66.4	56.4
Number of road intersections	323	295	277	266	251	236	211
Number of road endpoints	71	52	46	43	40	37	28

As shown in Table 3, the number of un-flooded road segments, intersections, and endpoints decreased along with increasing rainfall. More and more road segments were flooded when the return periods of rainfall increased. Under the 100a rainfall return period, the length of un-flooded roads accounted for only 36.24% of the original road network not affected by flooding. More than half the roads were flooded with a water depth above 0.15 m when the rainfall return period of the area reached 100a. Moreover, in Fig. 9, note that roads in the southern part of the case area were more affected by floods because un-flooded roads significantly decreased as rainfall increased. This is consistent with the flooding hotspots discussed in “Changes in flooded areas under various rainfall scenarios” section.

### Changes in road network connectivity

Connectivity changes in un-flooded road networks are shown in Table 4. As shown in the table, the values of *LNR*, *ID*, *RND*, and *RNCI* decreased as rainfall increased. The *RNCI* of the case area was only 0.57 during the 100a rainfall return period, which shows that more than 40% of road connectivity can be lost because of heavy storm flooding. This explicitly demonstrates the extent to which storm rainfall can reduce urban road connectivity in the case area.

**Table 4:** Changes in road network connectivity across various rainfall events.

Index of road network connectivity	Original road network	Un-flooded road segments under different return periods of rainfall					
		2a	5a	10a	20a	50a	100a
*LNR*	1.30	1.12	1.06	1.03	**0.98**	0.95	0.91
*ID*	7.52	6.86	6.44	6.19	**5.84**	5.49	4.91
*RND*	3.62	2.46	2.12	1.9	**1.7**	1.54	1.31
*RNCI*	1.00	0.82	0.75	0.71	**0.67**	0.63	0.57

Significant values are in [bold].

**Figure 10:**
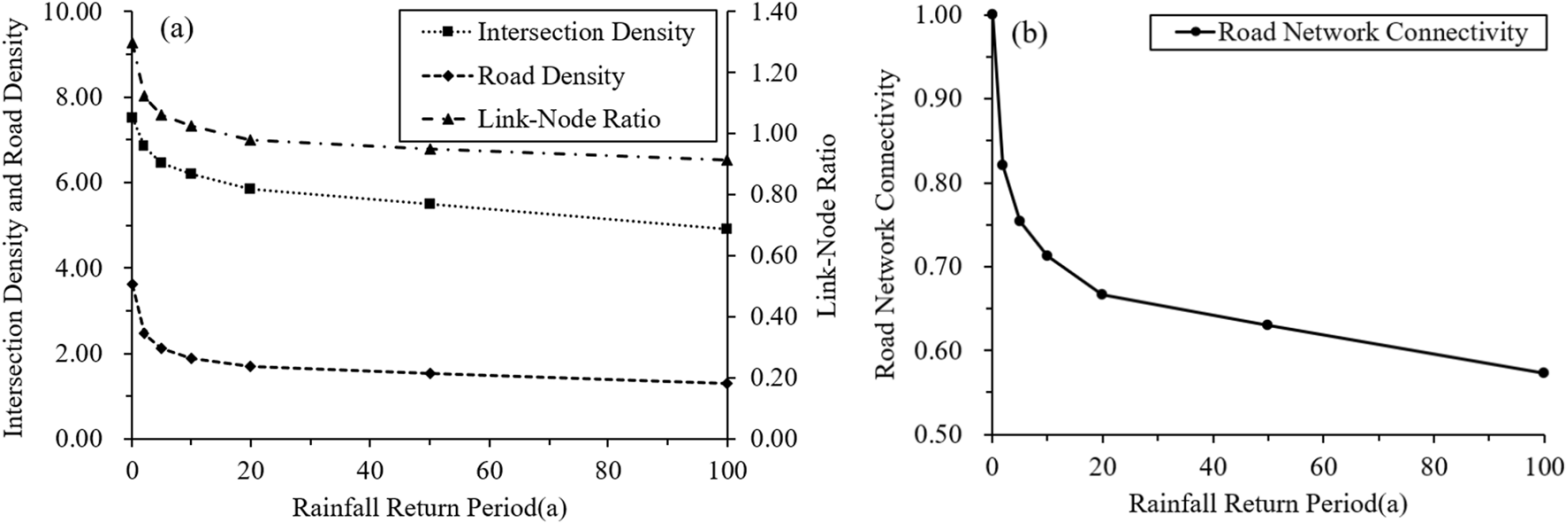
Relationship between road connectivity and rainfall. This graph was generated with OriginPro 2021 (Learning Edition)(https://www.originlab.com/).

Figure 10 shows the relationship between the road connectivity index and the rainfall return periods. Observe that the declining trend of *LNR*, *ID*, and *RNCI* tended to stabilize after the rainfall return period exceeds 20a. The *RNCI* in the case area declined to 0.67 in the 20a rainfall return period. This showed that the rainfall return period of 20a was a critical point in reducing road connectivity of the case area. For rainfall events in which the return period was less than this critical point, such as rainfall of 2a, 5a, and 10a, road connectivity declined more dramatically as rainfall increased, and thus, more adaptive traffic management is required to address the resulting flooding. This also implies that floods of moderate amounts such as those of 10a and 20a are more critical in influencing road connectivity in the case area and thus require more attention.

## Discussion

This study assesses changes in road network connectivity caused by urban floods of various rainfall intensities through urban hydrological simulation using MIKE Urban and MIKE 21 in a case area of southern China. Flood maps of the study area with inundated segments and water depths were generated via GIS technologies. The network connectivity of un-flooded roads was then calculated through three indicators: the *LNR*, *ID*, and *RND* to explore the impacts of urban floods on urban traffic. However, urban flood simulation is quite challenging as flood processes are affected by numerous factors including climate, topography, hydraulics, land use, and urban infrastructure. The impacts of topography and drainage networks on urban flood modeling are discussed to provide more detailed implications on the nature of flood processes in urban areas.

### Impacts of geographic and hydraulic factors on urban floods

#### Impacts of the micro-terrain in urban areas

Compared with catchment hydrologic modeling in mountain areas, urban hydrologic simulation is more challenging due to the requirement for micro-topographic data to include the strong spatial heterogeneity of urban land surfaces intensively manipulated by human activities. Thus, we employ a DEM image with a high resolution of 5 m from massive on-site measured elevation data at 147,640 points in the case area. These high-resolution DEM data play an important role in increasing the accuracy of urban hydrologic modeling, although such detailed data are not always available in many cities.

**Figure 11:**
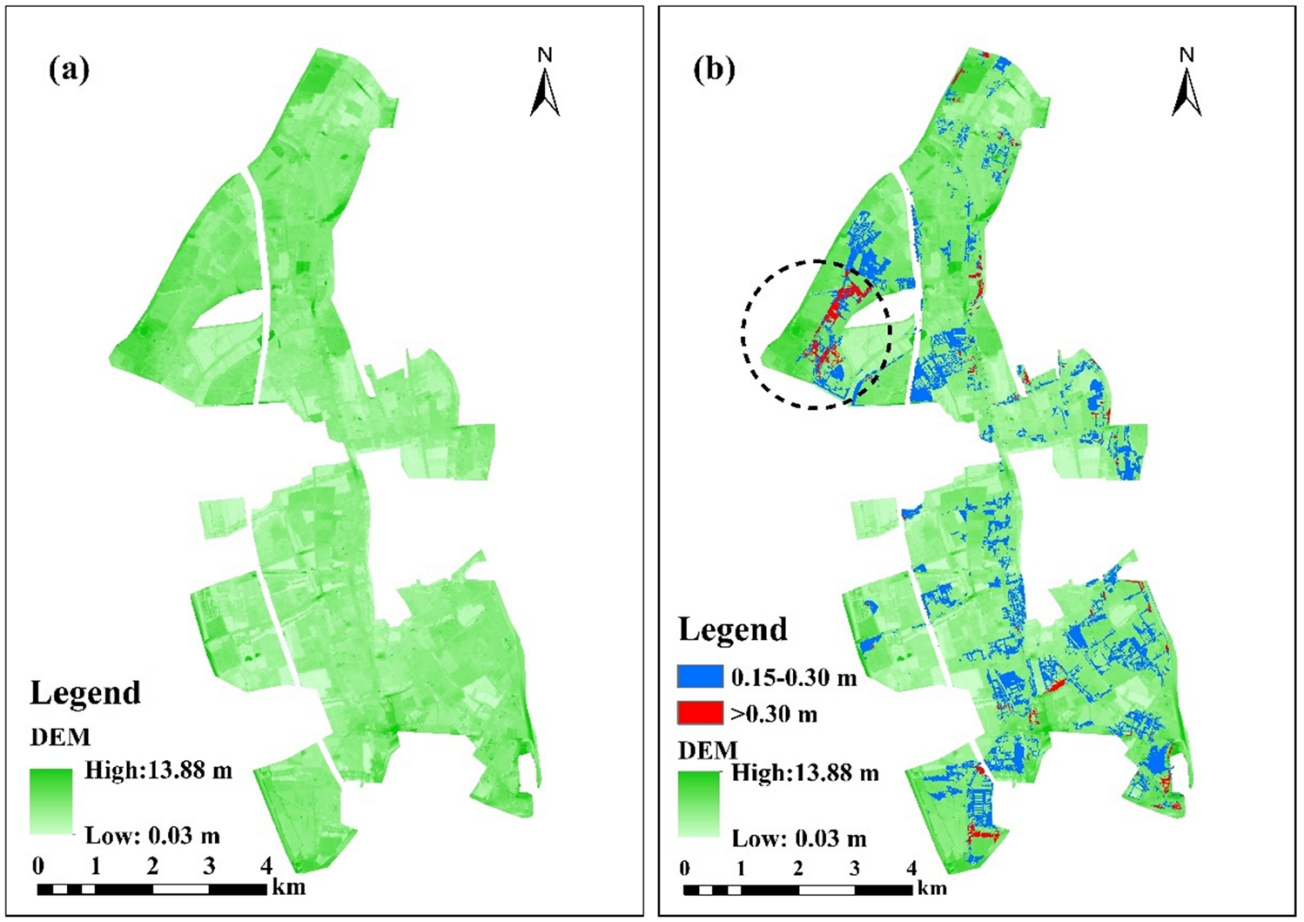
DEM and inundated areas of the case region: (**a**) DEM data; (**b**) inundated areas of the DEM image. This map was generated with ArcMap Version 10.0. (https://www.esri.com/en-us/arcgis/products/arcgis-desktop/overview).

Figure 11 shows the DEM data of the case area and the inundated areas with water depths during the 100a rainfall return period. Note that areas that are deeply inundated, such as the area marked by the dotted circle in the figure, are generally located in low-lying regions surrounded by higher objects, such as buildings or highways. This demonstrates that the locations of storm-induced waterlogging areas are not only related to the low-lying terrain of the area but are also affected by the higher elevations of surrounding regions, which could accelerate surface flow from the higher region to its adjacent low-lying spot, resulting in more severe flooding. These were supported by the study of Nobre et al.^58^, which developed a static approach for mapping the potential extent of inundation using the height above the nearest drainage terrain. They found the relative vertical distances from the higher spots to the nearest river is an effective distributed predictor of flood potential.

**Figure 12:**
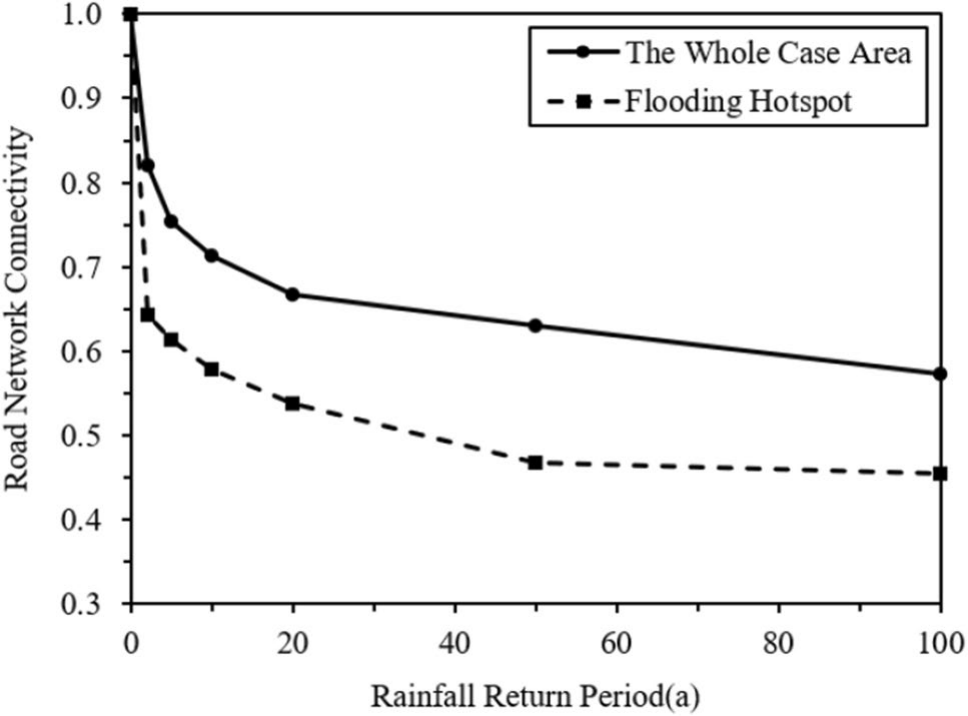
Relationship between road connectivity and rainfall in the flooding hotspot of the case area. This graph was generated with OriginPro 2021 (Learning Edition)(https://www.originlab.com/).

The decrease of road connectivity in the flooding hotspot which are circled by the dotted line in the Fig.11 are shown in Fig.12. The road connectivity in this area during the rainfall event was much smaller than that of the whole case area. Nearly 50% of the road connectivity was lost during the rainfall of 100-year return period. The micro-terrain feature of this area which can be recognized by the high-resolution DEM data had a significant impact on the modeling of road connectivity in the context of urban floods.

**Figure 13:**
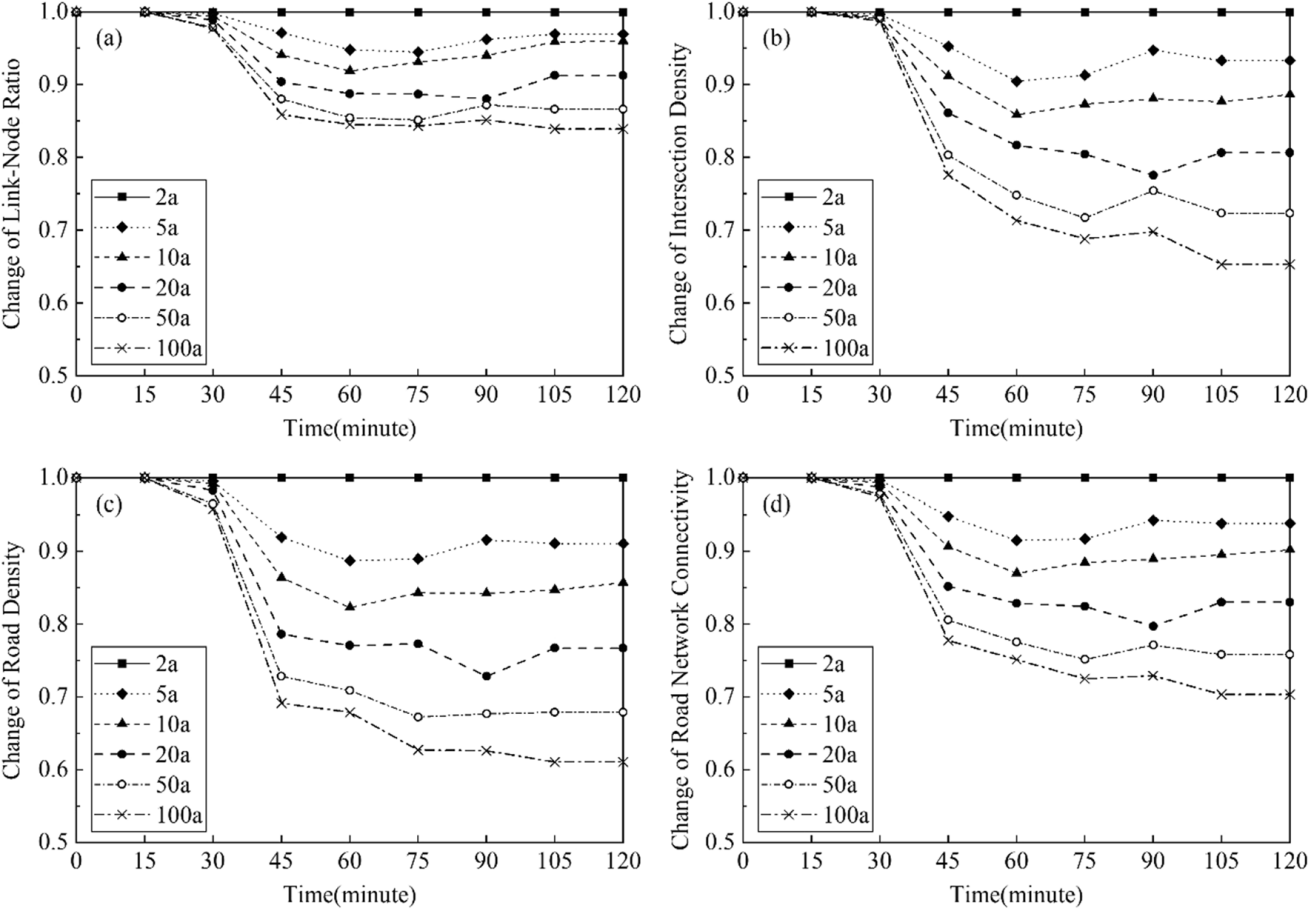
Changes of road connectivity in the case area: (**a**) Link-Node Ratio; (**b**) Intersection Density; (**c**) Road Density; (**d**) Road Network Connectivity. This graph was generated with OriginPro 2021 (Learning Edition)(https://www.originlab.com/).

Figure 13 show the changes of road network connectivity in the case area during the first two hours of the rainfall events with various given return periods. The changes of *LNR*, *ID*, *RND*, and *RNCI* under the rainfalls from 5 to 100a return periods were calculated by those indicator’s relative values to the 2a rainfall. Therefore, the values of indicators under the rainfall of 2a return period were 1.0 in the figures. As shown in the Fig. 13d, the road connectivity decreased and reached the lowest point until 75–105 min after the rainfall began. In addition, at the end of the rainfall event with the return period of 100a, the road connectivity of the case area was 70% of the connectivity at the same time during the 2a rainfall. Moreover, as shown in the Fig. 13a–c, the impacts of the urban flood on reducing the Intersection Density (*ID*) and the Road Network Density (*RND*) of the road network were larger than the impact on reducing the Link-Node Ratio (*LNR*). The *ID* and *RND* were reduced to approximately 60% during the 100a rainfall comparing with the 2a rainfall, while the *LNR* was only reduced to 85%. This means that the number of flooded roads and the number of flooded intersections were increased simultaneously when the rainfall increased, casing the similar extents of decreases of *ID* and *RND* in the un-flooded road network. This also led to a relatively smaller reduction of *LNR*, which was the ratio of the number of roads and the number of intersections in the un-flooded road network.

**Figure 14:**
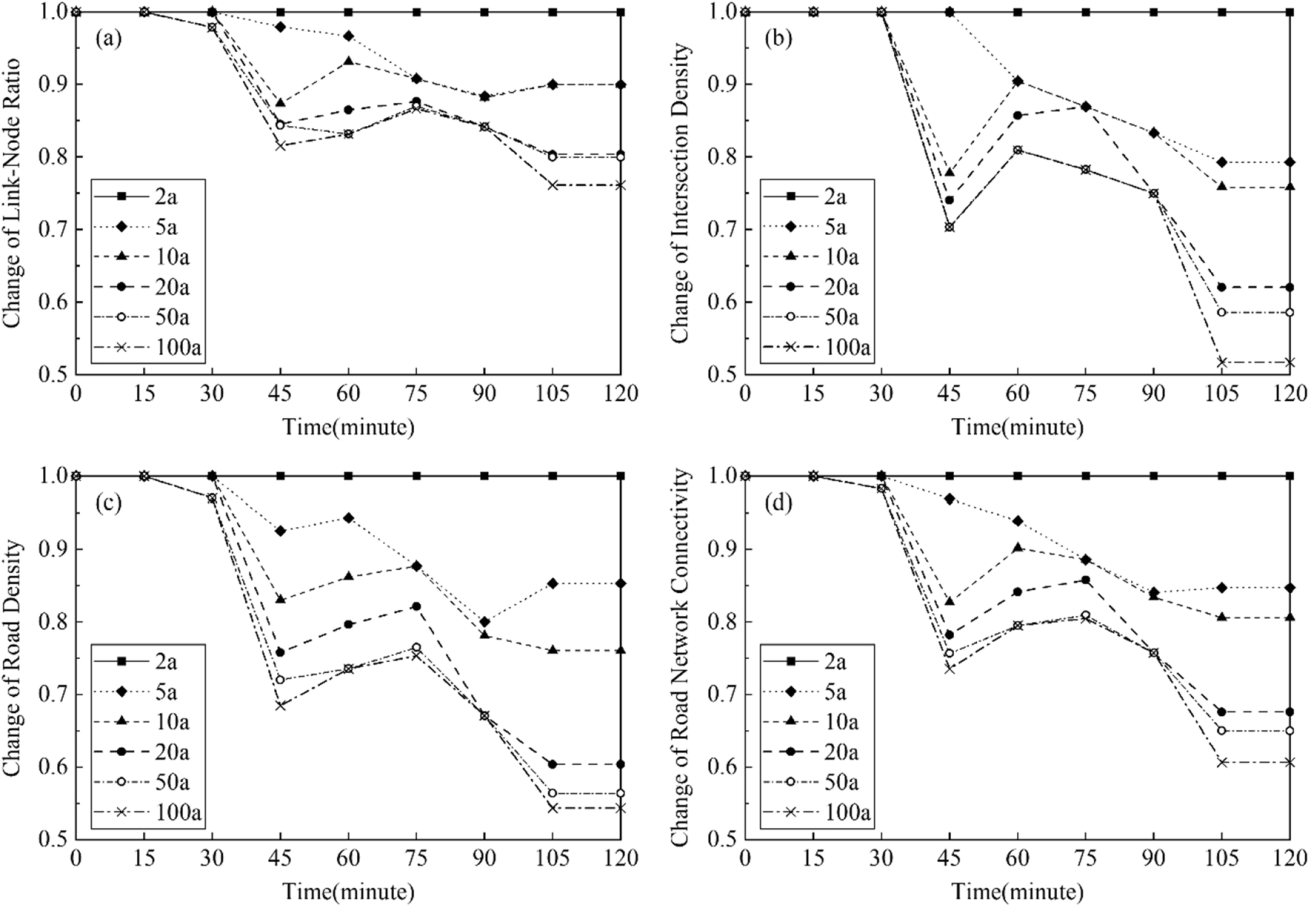
Changes of road connectivity in the hotspot of the case area: (**a**) Link-Node Ratio; (**b**) Intersection Density; (**c**) Road Density; (**d**) Road Network Connectivity. This graph was generated with OriginPro 2021 (Learning Edition)(https://www.originlab.com/).

Figure 14 show the changes of road network connectivity in the flooding hotspot of the case area. Comparing with the changes of *LNR*, *ID*, *RND*, and *RNCI* in whole case area (Fig. 13), the changes of these indicators in the flooding hotspot were larger, showing a higher level of the flood impact in this spot. For example, at the end of the rainfall event with the return period of 100a, the road connectivity at this spot was only 60% of the connectivity at the same time during the 2a rainfall. Moreover, there were sharper declines of the road connectivity during the rainfall events at the flooding hotspot, comparing with the reductions of the road connectivity in the whole case area. This means that the roads and intersections in this spot were flooded faster, thus requiring more timely and rapid response measures for the flood control.

#### Impacts of urban drainage networks

**Figure 15:**
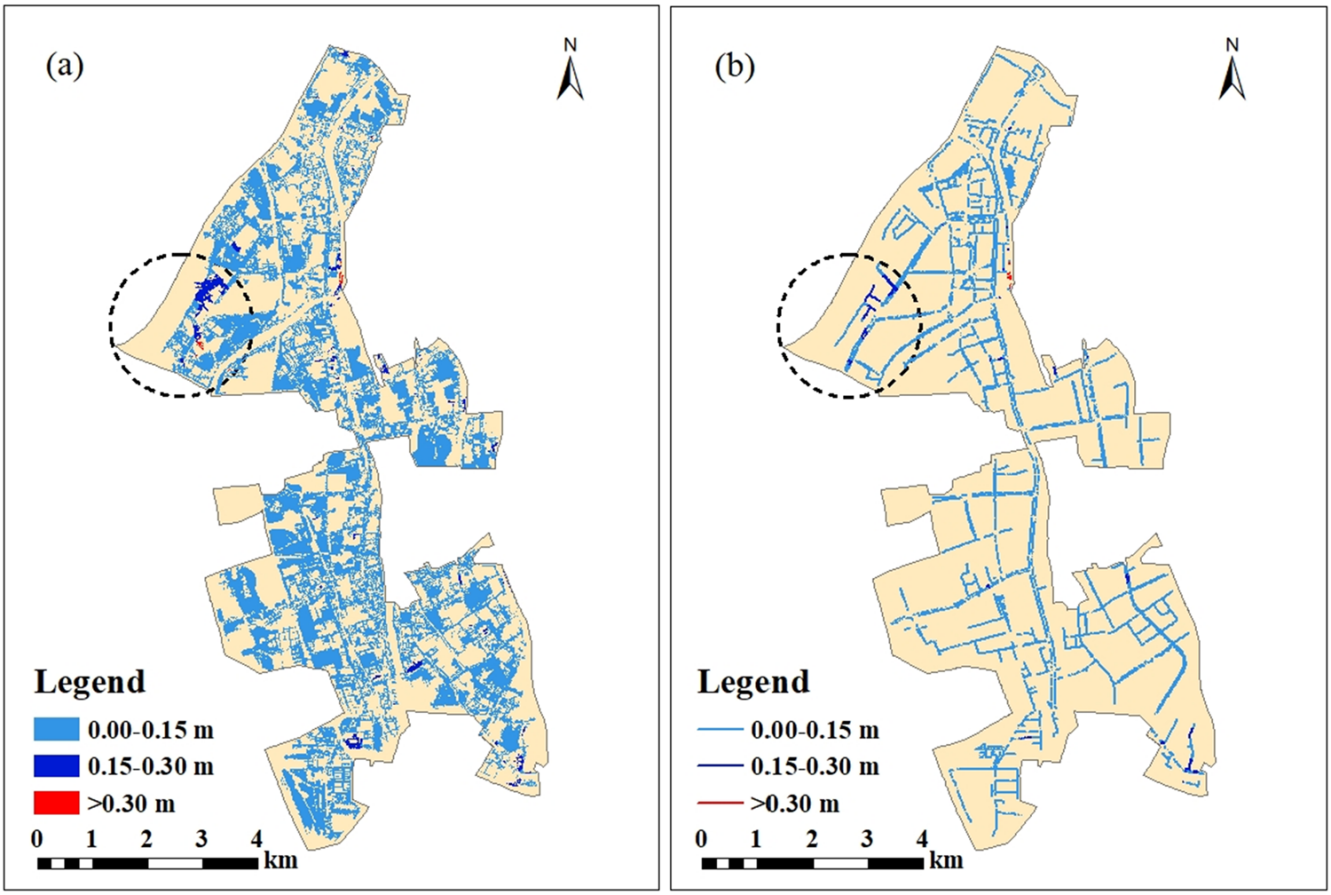
Distribution of (**a**) inundated areas and (**b**) pipelines where the differences in water depth between 2 and 100a rainfalls are greater than 0.15 m. This map was generated with ArcMap Version 10.0. (https://www.esri.com/en-us/arcgis/products/arcgis-desktop/overview).

Another challenge of urban flood modeling is the requirement for data on underground drainage pipelines, including pipeline maps, elevations, and diameters, to calculate the amount of water discharged from the surface into the ground. This study considered 5635 pipelines with a total length of 198 km, 5487 nodes, and 269 drainage outlets for a 1-D hydraulic simulation of the drainage process. The impacts of the underground drainage network on urban floods were explored by comparing the diameters of pipelines in areas with different inundated water depths in the context of various rainfall scenarios. For this, the differences of inundated water depths between 2a rainfall and 100a rainfall were calculated and divided into three grades, including 0.00–0.15 m, 0.15–0.30 m, and beyond 0.30 m. Within each grade, the areas and their underground pipelines corresponding to those water depth differences are depicted in Fig. 15. The length and average diameter of these pipelines are shown in Table 5.

**Table 5:** Differences in water depth between 2 and 100a rainfalls.

Difference in water depths between 2 and 100a rainfalls	0.00–0.15 m	0.15–0.30 m	> 0.30 m
Inundated area (km^2^)	16.72	0.49	0.02
Length of drainage pipelines in the inundated area (km)	184.41	6.37	0.40
Average diameter of the pipelines (m)	0.676	0.676	0.679

As shown in Fig. 15, areas with water depth differences over 0.15 m and 0.30 m were concentrated in the northwest part of the case region, which was a hotspot significantly affected by flooding as rainfall increases. However, the average diameter of the pipelines in this area was 0.676 m, the same as pipelines in other areas, as shown in Table 5. This could be a major limitation on flood control in this hotspot as the pipelines were as small as in other areas not significantly affected by floods. More engineering measures such as new drainage pipelines with larger diameter are required in this area to improve its drainage capacity. This is consistent with recent flood control initiatives of the local government, which regards this area as the one of the most vulnerable spots for flooding across the whole city. The local government invested more than RMB 6 million on updating drainage pipelines in 2017^59^.

### Challenges of model calibration and validation for urban flood simulation

Model calibration and validation are another major challenge in urban hydrological simulation. It directly affects the accuracy and applicability of models. Generally, gauged water levels and discharges in river courses are used to calibrate and verify the hydrological model. However, these data are not always available in many cities as there are insufficient hydrological gauge stations in urban areas, which leads to difficulties in urban hydrological simulation.

Previous studies used quantitative and qualitative methods to calibrate and verify urban hydrological models^60–62^. The data adopted for quantitative methods of calibration consist of gauged discharges at the outlets of the drainage pipelines^63^, gauged water level and river runoff^64^, and measured flood depth^65,66^. However, these gauged data are difficult to obtain, especially real-time flood depths in urban areas during rainfall events, as workable methods of measuring flood depth across a large urban area are currently rare. Qualitative methods verify urban hydrological models through comparing the locations of high flood-depth points calculated by models with flood hotspots identified by the local government’s experience or field surveys^67,68^. Empirical locations of flood hotspots are relatively easy to obtain. However, the amount of such data is small, especially long-term time series data. Consequently, it is nearly impossible to quantitatively calibrate model parameters using these location data. In any case, the costs of calibration and verification of urban hydrological models are substantial due to the limited gauge data. More efficient methods are required to obtain more data for model calibration and verification.

In this study, model verification uses data on flood depth obtained by on-site measurement using rulers and photo-based estimations. In addition to manually measuring water depth at various sites during rainfall events, flood photos and videos taken by volunteers were adopted to estimate the water depth. Several volunteers, including convenience store attendants and factory security guards, were recruited to photograph floods in their surrounding area during a selected rainfall event, using their smartphones. Photo shooting time and the GPS coordinates of the shooting location were also recorded and sent to the study authors. These pictures and videos were then used to estimate the water depth at these locations.

**Figure 16:**
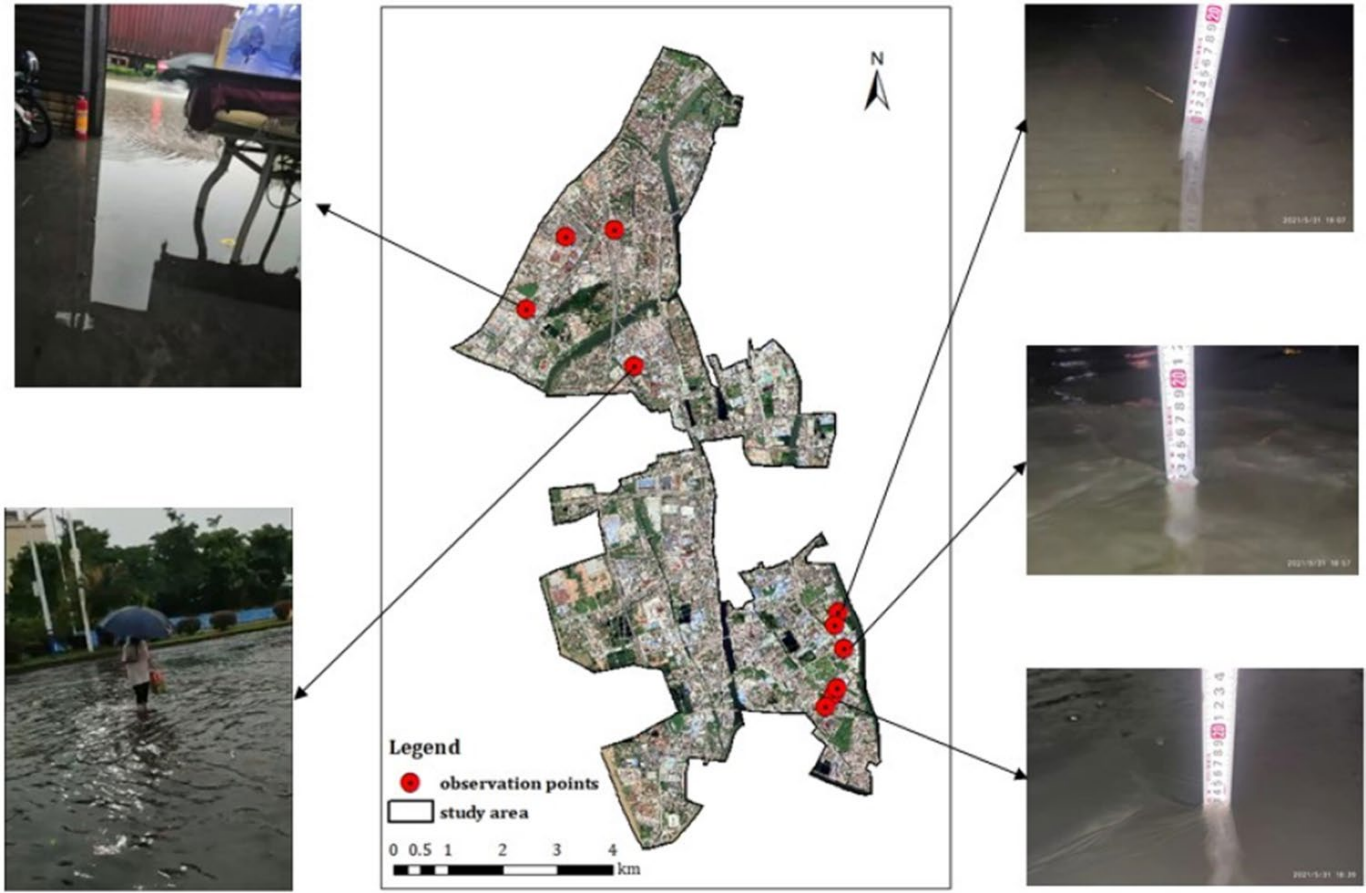
Measurement points and photos of flooding in the case area. This map was generated with ArcMap Version 10.0. (https://www.esri.com/en-us/arcgis/products/arcgis-desktop/overview).

The rainfall event in the case area from 17:00 to 22:00 on May 31, 2021 was selected for model verification. Maximum water depths during the rainfall at 10 locations were measured and estimated. Although only limited water depth data were obtained due to high labor requirements and costs of measurement, this provided a new method to estimate urban flood water depth in the context of citizen science. Citizens sharing their local situation in real time is becoming a rich source of data in the era of smart phones and the Internet^69^. Measurement points and photos of flooding are shown in Fig. 16.

## Conclusion

This study assesses changes in road network connectivity caused by urban floods under various rainfall intensities through urban hydrological simulation using MIKE Urban and MIKE 21 in a case area of southern China. Maps of flooded roads and their water depths are generated via GIS technologies. The network connectivity of un-flooded roads is then calculated using three indicators: the *LNR*, *ID*, and *RND* to explore the impacts of urban flooding on urban traffic.

Results indicate that road network connectivity decreases as rainfall increases. The negative relationship between road connectivity and rainfall return period demonstrates the impact of urban storm floods on road traffic. Moreover, a threshold of rainfall increases reducing road network connectivity is identified in the form of the rainfall return period. For rainfalls with a return period less than 20a, road network connectivity of the case area will be significantly affected by floods. In that case, more than 30% of road connectivity will be lost in the case area. For rainfall events with a return period less than 20a, road connectivity will decline more dramatically as rainfall rises, and thus, more adaptive traffic management is required to address such storms in the case area.

In addition, two hotspots vulnerable to floods in the case area are identified by analyzing the impacts of micro-topography and urban drainage networks on urban flood processes. Our results can inform more adaptive strategies for local flood and traffic management in the case area. The methods established here for hydrological model validation are applicable in broader regions as alternatives to obtain richer data for more effective urban hydrological simulation.

## Data Availability

The data that support the finding of this study are available from the corresponding author upon reasonable request.
